# Successful introduction of cardiac index, fluid responsiveness and oxygen delivery data into the primary survey at a central London major trauma centre and impact on time to CT, fluid resuscitation and disposal

**DOI:** 10.1186/cc12139

**Published:** 2013-03-19

**Authors:** S Helyar, I De Abreu, S Holloway, P Hopkins

**Affiliations:** 1 King's Health Partners AHSC, London, UK

## Introduction

The use of cardiac output monitoring has been shown to be beneficial in the setting of perioperative medicine and critical illness [1,2]. More recently, its application in the setting of major trauma has been described [3]. Here, we describe our preliminary experience of embedding bioreactance flow monitoring within the major trauma primary survey of severely injured patients and the subsequent effect on patient management.

## Methods

Institutional ethical approval was obtained. Intubated major trauma patients were sequentially enrolled. Exclusions included major thoracic burns and children. Bioreactance flow monitoring (NICOM; Cheetah) was applied at the same time as ECG leads and the calibration step performed during handover from the prehospital team. Time to availability of oxygen delivery data was recorded and trauma team members surveyed regarding for perceived benefits and concerns from this monitoring. The influence of flow monitoring on fluid resuscitation, time to CT and definitive disposal (to OR/ICU) was measured and compared with a control population matched for injury severity score, age and sex.

## Results

Cardiac index was available at mean 10.6 minutes (median 9 minutes; SD 3.9), fluid responsiveness at mean 35.9 minutes (median 35; SD 11.3) and oxygen delivery calculation at mean 25.3 minutes (median 25; SD 7.7). Passive leg raise was not performed in 63% of patients due to concerns about pelvic or brain injury. Volume of fluid infused (mean 738 vs. 925 ml; *P *= 0.124), time to CT (mean 57.4 vs. 68.8 minutes; *P *= 0.08), and time to definitive disposal (mean 124.9 vs. 146.1 minute; *P *= 0.069) were all reduced in the flow monitored group, although not significantly different when compared with a matched control group (Mann-Whitney U rank sum). Eighty-four percent of trauma team members surveyed felt the flow monitoring data to be useful, and only 11% felt it may impair clinical management.

## Conclusion

Cardiac index, fluid responsiveness and oxygen delivery data can be obtained inform a primary survey. Rather than introducing delays, the use of flow monitoring was associated with a trend towards decreased time to imaging; less fluid use pre-damage control point and reduced time to definitive disposal. Further research is required to confirm benefits and mechanism.
